# Mode-resolved frequency comb interferometry for high-accuracy long distance measurement

**DOI:** 10.1038/srep14661

**Published:** 2015-09-30

**Authors:** Steven. A. van den Berg, Sjoerd van Eldik, Nandini Bhattacharya

**Affiliations:** 1 VSL, Thijsseweg 11, 2629 JA Delft, The Netherlands; 2 Technische Universiteit Delft, Lorentzweg 1, 2628 CJ Delft, The Netherlands

## Abstract

Optical frequency combs have developed into powerful tools for distance metrology. In this paper we demonstrate absolute long distance measurement using a single femtosecond frequency comb laser as a multi-wavelength source. By applying a high-resolution spectrometer based on a virtually imaged phased array, the frequency comb modes are resolved spectrally to the level of an individual mode. Having the frequency comb stabilized against an atomic clock, thousands of accurately known wavelengths are available for interferometry. From the spectrally resolved output of a Michelson interferometer a distance is derived. The presented measurement method combines spectral interferometry, white light interferometry and multi-wavelength interferometry in a single scheme. Comparison with a fringe counting laser interferometer shows an agreement within <10^−8^ for a distance of 50 m.

Since the invention of the femtosecond frequency comb at the beginning of this century^1^, a wealth of applications have been developed in rather distinct fields^2^,^3^,^4^. Primarily the femtosecond frequency comb has been applied to accomplish directly traceable measurement of optical frequencies to the SI second^5^. Here the frequency comb serves as a transfer oscillator that connects the relatively low frequencies that are available from atomic clocks, defining the second, to optical frequencies that are 4–5 orders of magnitude larger. Being able to bridge this gap in a single step has appeared to be a giant leap forward in the field of optical frequency measurement. This has not only strongly influenced the field of high-resolution optical spectroscopy, but also the field of length metrology. The primary standards that are used for length measurements are stabilized laser sources. The wavelength of these optical frequency standards serve as a ruler for length or distance measurement, which takes place via optical interferometry. Already since 1983 the meter has been formally connected to the SI second, by fixing the speed of light in vacuum *c* to 299 792 458 m/s by definition. As a result, the optical wavelength is calculated from the optical frequency, which thus relates the meter to the second. Before the invention of the frequency comb the calibration of an optical frequency standards was a tedious job, requiring a very complex ‘frequency chain’, which was costly, hard to operate and only suitable to reach a specific wavelength in the optical domain^6^. Via this link the wavelength of iodine-stabilized Helium-Neon (HeNe) lasers was connected to the SI second. However, due to the complexity of the chain the connection to the SI second was rather loose and the performance of the lasers was guaranteed in practise by regular mutual comparison and operation following a ‘Mise en Pratique’^7^. Iodine stabilized HeNe lasers are still widely used as primary frequency standards for dimensional metrology, serving as a reference standard for calibrating helium-neon based counting laser interferometers. Direct traceability to the SI second via the frequency comb is now common practise.

However, the frequency comb can also be applied as a measurement tool for distance metrology directly. One of the first schemes utilized the frequency comb as a source for frequency-modulation based distance measurement^8^. Subsequently, schemes based on cross-correlation measurement, utilizing the fixed pulse-to-pulse distance, have been proposed and demonstrated^9^,^10^,^11^. Distance measurement has also been shown using spectral interferometry^12^,^13^, multi-heterodyne interferometry^14^ and time-of-flight measurement^15^. In addition, comb-referenced multi-wavelength interferometry has been demonstrated as a useful tool for positioning and distance measurement^16^,^17^. A similar measurement scheme has also been applied for probing other dimensional quantities, e.g. surface profiles^18^,^19^.

In this paper we demonstrate distance measurement with a femtosecond frequency comb for distances up to 50 meter, based on frequency resolved comb interferometry^20^. The advantage of this method compared to multi-heterodyne interferometry is that only one frequency comb is needed. Furthermore the distances that can be measured are not limited to regions of overlapping pulses, which is the case for cross-correlation based distance measurements. A huge advantage of frequency comb interferometry compared to conventional interferometry with a single wavelength is that the range of non-ambiguity is determined by the pulse-to-pulse distance, which is typically tens of cm to meters. For a conventional single-wavelength interferometer the range of non-ambiguity is half of the optical wavelength. As a result these interferometer systems are usually used in fringe-counting mode, i.e. a displacement is measured instead of an absolute distance. Such an incremental measurement requires a linear guidance and uninterrupted beam path. Because of its long range of non-ambiguity this limitation is not encountered for the techniques using the frequency comb. In this work a comparison between the distance measured by the frequency comb and a counting interferometer will be presented. We have already demonstrated spectrally resolved comb interometry for a short displacement (up to 15 cm)^20^. Here a huge step is taken by demonstrating the power of mode-resolved comb interferometry for long range applications, up to 50 m in air, which is promising for a wealth of applications requiring high-accuracy and absolute long distance measurement.

## Measurement principles

**Figure 1:**
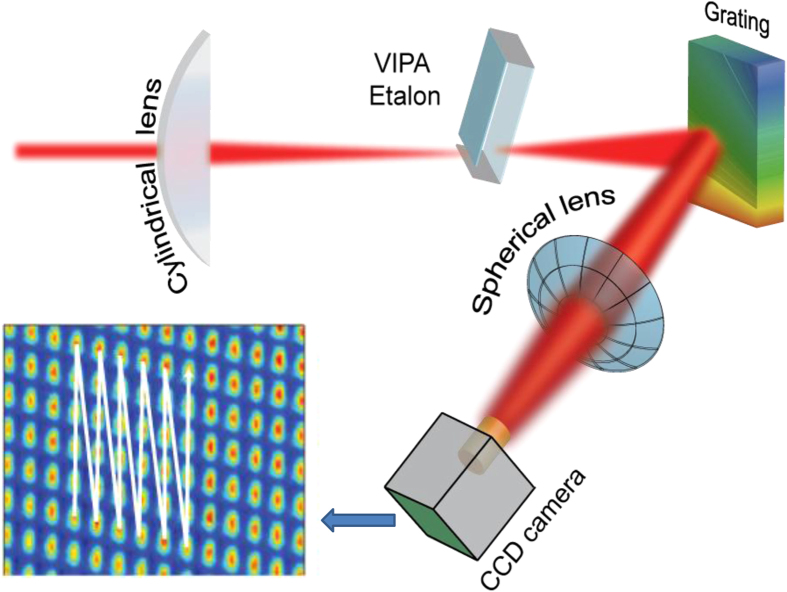
High-resolution spectrometer based on a VIPA and a grating for analysis of the frequency comb spectrum. The full comb spectrum is reconstructed by stitching vertical lines together, as indicated with the white arrow. For clarity only a small part of the full image is shown. In practice a vertical line contains about 50 unique dots.

A femtosecond frequency comb is the spectrum of a laser that is stabilized against a time standard like a cesium atomic clock, by phase-locking both the repetition rate *f*_*rep*_ and the offset frequency *f*_0_. As a result the spectrum of the comb consists of thousands of optical modes, with a fixed mutual frequency difference of neighboring modes equal to *f*_*rep*_. These frequencies have a stability comparable to the stability of the reference clock. For a commercial cesium clock this is typically 10^−11^ in 1 second. In the time-domain the stabilization of *f*_*rep*_ leads to the stabilization of the distance of the emitted pulses at the same level. In our measurement scheme, we send the light of a frequency comb into a Michelson interferometer. The interferometer output is subsequently analyzed with a high-resolution spectrometer based on a virtually imaged phase array (VIPA) and a grating. The VIPA, an etalon with high-reflectivity coatings, is a key component of the setup. The comb light is focused with a cylindrical lens on the lower uncoated part of the VIPA, which is slightly tilted. As a result different wavelengths are resonant at different angles of incidence and angular wavelength dispersion is generated^21^. The free spectral range of the VIPA is about 50 GHz in our case. In order to separate the overlapping orders of the VIPA output a grating is used. Finally the light is imaged onto a CCD (charge-coupled device)-camera. The spectrometer images that are acquired in this way consist of dots, which are resolved frequencies of the comb. A schematic overview of the measurement approach is shown in Fig. 1. From this image the comb spectrum is reconstructed by stitching vertical lines, as explained in more detail in the description of the experiment below. By resolving the comb spectrum, data for both spectral interferometry and homodyne interferometry are available. In the case of spectral interferometry the phase change as a function of wavelength is used to determine the distance. For homodyne interferometry the knowledge of each individual wavelength is used in addition.

### Spectral interferometry

With the repetition frequency being stabilized against the cesium atomic clock, an almost perfect frequency scale is available for determining the slope of the phase change with wavelength. In our case we determine this phase change with wavelength from a cosine fit through the measurement data, which is equivalent to unwrapping the phase by fast Fourier transform and determining the slope. The interference term can be written as:


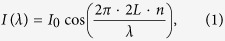


with *I*_0_ the intensity of the light sent into the interferometer, *L*, the path length difference of the interferometer arms (single path), *λ* the vacuum wavelength and *n* the refractive index of the medium, usually air. The total accumulated phase can be written as:


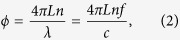


with *f* the optical frequency and *c* the speed of light in vacuum.

The phase change as a function of frequency can be written as:


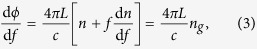


with *n*_*g*_ the group refractive index:


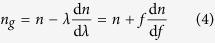


This leads to the following expression for the distance L:


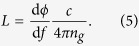


Note that if *n*_*g*_ depends on *λ*, i.e. if there is group velocity dispersion, d*ϕ*/d*f* is not constant. If group velocity dispersion cannot be neglected the approach based on cosine fitting, having a fixed periodicity, cannot be applied anymore, since some chirp will occur as a result from the nonlinearity. In the time domain this leads to a deformation of the pulse shape. For the distance that we measured and the targeted uncertainty we can neglect group velocity dispersion. This is equivalent to the assumption that the refractive index linearly depends on wavelength. It can easily be shown that in that case *n*_*g*_ is wavelength-independent. As demonstrated by M. Cui *et al.*^11^ the effect of group-velocity dispersion is far below 1 *μ*m for a 50 m distance, so this assumption is valid for our case.

In practice d*ϕ*/d*f* is obtained from the fitting a cosine through the data with *ϕ* = *C*(*p* − *D*). Here *C* and *D* are fitting parameters and *p* is a label associated to a specific comb frequency: *f*_*p*_ = *f*_*rep*_(*Q* − *p*) + *f*_0_, with *Q* a large integer number. As a result d*ϕ*/d*f* = −*C*/*f*_*rep*_. This leads to


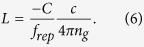


In the description of spectral interference we have given so far, the distance *L* is purely determined from spectral interferometry, which leads to solutions:


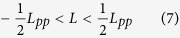


Here *L*_*pp*_ is the pulse-to-pulse distance in the medium, being defined as:


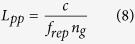


The fact that the range of *L* is restricted to the range as given in Eq. (7), is a direct result of the periodicity of the pulse train. Here the method is different from conventional white-light interferometry, as will be discussed later. Additional information is needed for the determination of the sign of *L*, since the sign of the parameter *C* cannot be determined from the cosine fit. There are several ways to obtain this information. For example, the sign can be retrieved from the observation of the change of the d*ϕ*/d*f* when moving the measurement arm towards the measurement position. Suppose that the measurement position is approached in the direction of increasing the interferometer arm length. When the fringe density decreases when approaching the measurement position *L*_*pp*_ is negative; for increasing fringe density *L*_*pp*_ is positive. Alternatively, one may change *f*_*rep*_ (and thus *L*_*pp*_) and observe the change of d*ϕ*/d*f*. A third possibility is that |*L*/2| exceeds the uncertainty of a rough measurement that is performed with another method. In that case the information from the rough measurement is sufficient to determine the sign of *L*.

Due to the periodicity of the pulse train, the interference patterns are repeating themselves. Obviously, pulse-overlap occurs when the total path length difference of the interferometer *L*_*t*_ (single path) is at a multiple of the pulse-to-pulse distance:


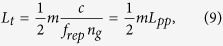


with *m* an integer.

For distance measurement based on spectral interferometry, the distance is determined from the d*ϕ*/d*f*, which equals 0 at distances that are multiples of *L*_*pp*_. This hold in vacuum and also in very good approximation in linearly dispersive media (within 10^−9^). An arbitrary distance is written as:


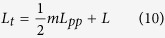


To determine *L*_*t*_, the integer *m* needs to be known. Therefore it is necessary to know the distance to be measured with an accuracy better than *L*_*pp*_/2. This is a relaxed requirement, with *L*_*pp*_ ≈ 30 cm for our system, that can easily be fulfilled by measuring the approximate distance with a simple electronic distance meter, time of flight measurement or even a measurement tape. Furthermore it is necessary to determine the sign of *L*, as described above.

### Homodyne many-wavelength interferometry

Once a distance has been determined using spectral interferometry, the knowledge of the individual wavelengths can be exploited. The cosine fit through the data also provides the phase of each individual wavelength. For homodyne interferometry this phase is usually determined from the relative intensity of the interference fringe, but in this case we determine it from the relative position of the wavelength on the cosine fit. In this way the measurement is not sensitive to potential intensity variations. The distance can then be determined for a particular wavelength *λ*_*p*_ via


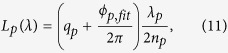


with *q*_*p*_ an integer number, and *n*_*p*_, the (phase) refractive index of *λ*_*p*_. The phase corresponding to the wavelength *λ*_*p*_, *ϕ*_*p*, *fit*_ is parametrized by the fitting parameters and is described by *ϕ*_*p*,*fit*_ = *C*(*p* − *D*) mod 2*π*. The integer number of wavelengths *q*_*p*_ is determined from the distance information obtained from the spectral interferometry result, which is accurate enough to provide a resolution within an optical fringe.

### The measurement process in 3 steps

To summarize, the total distance determination consists of 3 steps. First a rough pre-measurement is performed to determine the distance within a fraction of the pulse-to-pulse distance *L*_*pp*_. Secondly, the distance is determined more accurately based on spectral interferometry. By rewriting Eq. (6) to: *L* = −*C*/2*π* ⋅ Λ/2*n*_*g*_ and combining with Eq. (10) one obtains the following equation describing distance determination based on spectral interferometry:


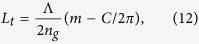


with Λ = *c*/*f*_*rep*_ the (vacuum) synthetic wavelength of neighboring comb modes, related to *L*_*pp*_ via *L*_*pp*_ = Λ/*n*_*g*_. The parameter *C* is negative for *L* > 0, as discussed above and *m* is an integer number as determined from the the rough measurement.

The third step is to determine the distance by using the additional information of the accurately known optical wavelengths in the combs, giving


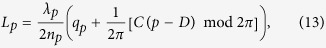


which is obtained by combining Eq. (11) with the parametrized phase *ϕ*_*p*_ = *C*(*p* − *D*) mod 2*π*. Note that *q*_*p*_ ≫ *m*, since *λ*_*p*_ ≪ Λ. As a result the measurement uncertainty resulting from Eq. (13) is expected to be smaller than the measurement uncertainty resulting from Eq. (12), since the uncertainty is mainly determined by the uncertainty on the fitting parameters *C* and *D*. For a practical measurement other contributions to the measurement uncertainty arise, like wavelength stability and, dominantly in our case, the stability of the interferometer itself.

**Figure 2:**
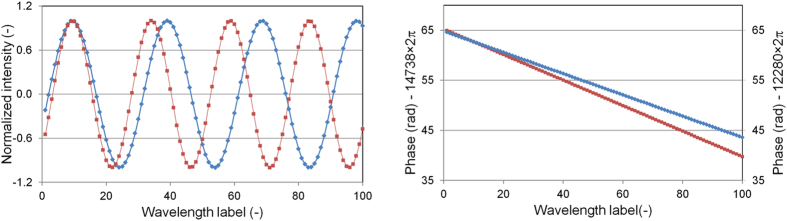
Simulated spectral interferometry for a distance L equal to 5 mm (blue) and 6 mm (red) respectively. The left graphs shows the simulated intensity for these delays, the right graph the phase. The absolute phase (4*πL*/*λ*) has been offset by an arbitrary multiple of 2*π* for both distances, as indicated on the axes. For clarity only 100 wavelengths are shown, corresponding to a range of 813.65 to 813.87 nm. Both the slope of the phase with wavelength and the absolute phase at a particular wavelength are used for determining absolute distance. Note that at zero delay the absolute phase would equal zero for all wavelengths.

Both methods are illustrated in Fig. 2, showing a simulation of interference and the phase change as a function of wavelength for delays of 5 and 6 mm, respectively. For spectral interferometry only the slope of the phase with wavelength is exploited, whereas for homodyne interferometry the absolute phase difference for a particular wavelength is utilized.

## Results

**Figure 3:**
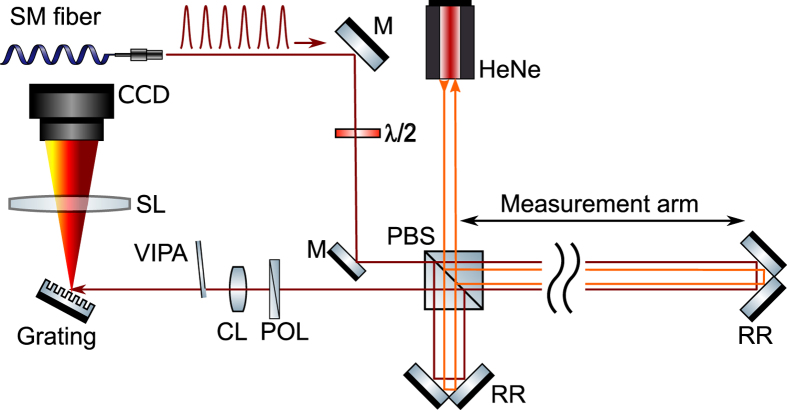
Schematic overview of the measurement setup for comparing distance measurement up to 50 m with a counting helium-neon laser and a frequency comb. The comb light is delivered to the setup with a single mode (SM) fiber, providing a clean mode profile. Both the HeNe laser (orange line) and comb laser (red line) measure the displacement quasi-simultaneously. PBS: polarizing beam splitter, POL: polarizer, *λ*/2: half-waveplate, CCD: charge-coupled device camera, M: planar mirror, RR: hollow retro reflector, CL: cylindrical lens (100 mm focal length), SL: spherical lens (400 mm focal length).

We exploit a Ti:Sapphire laser as a frequency comb for the experiment. The laser operates at a repetition frequency of 1 GHz. About 6000 wavelengths in the range from 813.5 nm to 827.5 nm are used for the experiment. This corresponds to *Q* = 363664, *p* = 1…6000. The frequency comb is phase-locked to a cesium atomic clock with a stability of 10^−11^ in 1 second. About 10 mW of optical power is provided to the experiment via a single mode fiber. The light is sent into a Michelson interferometer, which is polarization sensitive by using a polarizing beam splitter for directing the light into the measurement and reference arm. The light is reflected by gold-coated hollow retroreflectors. The polarization of the incoming comb light is controlled with a *λ*/2 plate. We have chosen for this configuration because it allows for direct comparison to an independent laser interferometer, which is a heterodyne system with orthogonally polarized modes, requiring a polarization sensitive interferometer. By using the same interferometer with both lasers, effects of vibrations and drift on the comparison are minimized. After the Michelson interferometer, interference between the orthogonal components of the frequency comb light is obtained by a polarizer inserted at 45°. The light is subsequently analyzed with a spectrometer based on a Virtually Imaged Phase Array (VIPA) spectrometer, which is inspired on VIPA applications in telecommunications^21^ and comb spectroscopy^22^. The VIPA has a free spectral range (FSR) of 50 GHz and coating reflectances of >99.94% and 99.5%, respectively. The VIPA provides angular dispersion along the vertical axis. A grating (blazed, 1200 grooves/mm), provides angular dispersion in the horizontal plane. The light is imaged on charge-coupled device (CCD) camera with a 400 mm lens, resulting in individually resolved comb wavelengths, appearing as dots. An overview of the setup is shown in Fig. 3.

In order to stitch the data correctly, it is necessary to identify the wavelength of individual dots. For this purpose we have coupled a tunable single-mode distributed feedback (DFB) diode laser, operating around 817 nm, into the same fiber that delivers the frequency comb light to the experiment. In parallel, the wavelength of the DFB-laser is measured with a wavemeter with an absolute accuracy of several tens of MHz. The laser thus provides a known reference frequency to the VIPA spectrometer, which is recorded by the CCD camera as a single dot (or sometimes 2 dots, because of the finite FSR of the VIPA). In this way, several reference frequencies are generated within the tuning range of the DFB laser (≈1 nm). Based on these reference markers and the values of *f*_*rep*_ and *f*_0_, the absolute wavelength of each dot can be determined. Each vertical line contains about 50 unique dots, as determined by the FSR of the VIPA. The full spectrum is reconstructed by stitching these lines using the information of the calibration procedure as described above. The resolution of the VIPA spectrometer as defined by the FWHM of an individual dot is approximately 0.55 GHz, based on a FWHM of a dot of 11 pixels and a separation of neighbouring dots of 20 pixels on the CCD camera. This is sufficient to resolve a comb with a repetition rate of 1 GHz.

The length of the measurement arm of the Michelson interferometer can be changed over a distance of 50 m, by moving a carriage along a straight linear guidance. We have measured distances up to 50 m in steps of 5 m. Here the position of the carriage was chosen arbitrarily, but regions with very few fringes (almost overlapping pulses) and very many fringes (pulses almost at maximum separation), were avoided to allow for a good fit of the data. For each measurement point the reading of the counting interferometer and frequency comb measurement are compared. Both measurements are performed quasi-simultaneously. The accumulation time of the camera was set at 1–2 ms. For such acquisition time good fringe contrast pictures were obtained. At longer accumulation time the interference fringes start losing contrast due to limited interferometer stability (vibrations, air turbulance). For each position 5 measurements have been taken. Simultaneously, the environmental conditions have been measured. Air pressure and humidity are measured at one position and the temperature is measured every 10 m along the measurement bench. Based on these measurements the refractive index of air is determined following the updated Edlén formula^23^.

### VIPA measurement results

**Figure 4:**
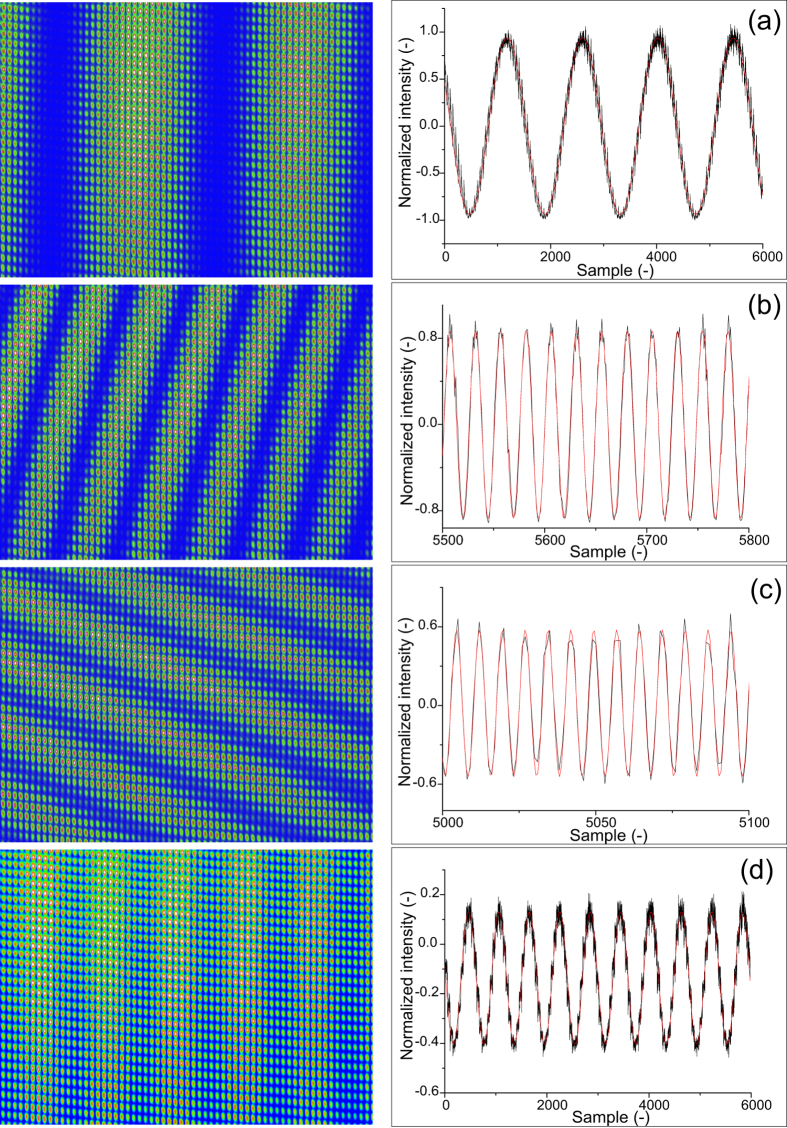
Spectral interference as measured with the VIPA spectrometer for distances of ≈0 m (a), 5 mm (b), 20 m (c) and 50 m (d), respectively. Spectral interferometry images are shown on the left. For clarity only 1/4 of the CCD chip area has been selected. On the right side the reconstructed spectra are shown. The *x*-axis with the sample number, has been scaled differently for these graphs to clearly visualize the fringes.

**Figure 5:**
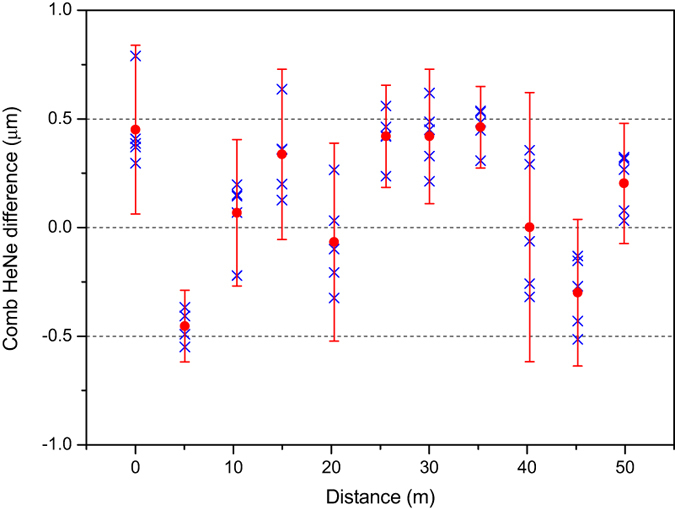
Observed differences between the frequency comb distance measurement, based on spectral interferometry and the counting laser interferometer. The error bars indicate twice the standard deviation over the 5 measurements.

In Fig. 4 typical VIPA CCD-camera pictures are shown, for an interferometer length of 0 m, 5 mm, 20 m and 50 m, respectively. The stitched data are shown on the right-hand side. The data have been normalized to the envelope of the comb spectrum using the spectral data as obtained from both the measurement and the reference arm individually. Based on the curve fit and the determination of the integer number of pulses and the sign, a distance is derived based on the spectral interferometry method for each position following Eqs (10) and (6). Figure 5 shows the measured differences between the counting laser interferometer and the comb-measurement. The agreement between both methods is within 1 *μ*m over the full length of the measurement bench for each individual measurement and <500 nm when averaged over 5 measurements. At a distance of 50 m this is a relative agreement within 10^−8^.

## Discussion

Remarkably, the agreement between the measurements with HeNe laser and frequency comb and the corresponding standard deviation are independent from the distance that is measured. This is explained by environmental vibrations that are coupled to the moving carriage of the 50 m measurement bench, dominating the measurement uncertainty, independent from the position of the carriage on the guidance. We have analyzed the measurement data to derive a distance based on homodyne interferometry as well, following Eq. (13). As worked out in the “Methods” section the measurement results based on homodyne interferometry are expected to be much more accurate than those based on spectral interferometry, because the uncertainty contribution to the phase-determination scales with *λ* instead of Λ. However, from our analysis of the homodyne measurements it appears that results are very similar for these measurements. Both methods agree with each other within 100 nm for all distances. The expected superior performance of homodyne interferometry is hidden by the mentioned environmental vibrations that dominate the measurement uncertainty of this comparison. The total estimated measurement uncertainty for a coverage factor *k* = 2 (corresponding to a 95% coverage interval) is 0.75 *μ*m for spectral interferometry, against 0.69 *μ*m for homodyne interferometry. This is in good agreement with the observations in Fig. 5.

As already discussed in the section “Measurement principles”, the patterns as shown in Fig. 4, repeat themselves every Λ/2. Here the method differs from real white light interferometry with a continuous spectrum. With conventional white light interferometry fringes get closer and closer for increasing path length difference. The resolution of the spectrometer determines the maximum distance that can be measured. However, since the spectrum of a comb is discrete, at some point a case of under sampling, or aliasing occurs. This happens for path length differences exceeding *L*_*pp*_/2. When *L* = *L*_*pp*_/2 the pulses have maximum separation in the time domain (corresponding to a phase difference of *π* between neighboring modes). The Nyquist frequency is exceeded when the pulse separation is further increased and the comb doesn’t contain enough frequency components (samples) that would be needed to map the (continuous) white light interference spectrum. In fact, we profit here from the fact that the spectrum is discrete and aliasing occurs, since the measurement range is now no longer limited by the resolution of our spectrometer as would be the case with a continuous spectrum. In the time domain this can be understood from the fact that a pulse train is used, with the result that the maximum distance between pulses arriving at the spectrometer output will never exceed *L*_*pp*_/2.

It should be noted that for absolute distance measurement in air, the knowledge of the refractive index becomes a limiting factor. Having an uncertainty on the Edlén formula of 2 × 10^−8^ plus additional contributions resulting from the measurement uncertainty of the environmental parameters, this is typically 10^−7^. The fact that the agreement between both methods can be demonstrated with higher accuracy, results from the fact that the contribution of the refractive index to the measurement uncertainty on the comparison largely cancels. Recently, promising frequency comb based techniques air refractive index compensation techniques have been reported, without the need for accurate measurement of environmental parameters^24^.

## Conclusion

We have demonstrated absolute distance measurement for distances up to 50 m utilizing thousands of wavelengths from a femtosecond frequency comb, resolved by a VIPA spectrometer. The range of non-ambiguity is given by the pulse-to-pulse distance of the laser, which is typically of the order of decimeters to meters, putting relaxed requirements on the initial knowledge of the distance to be measured. Therefore this measurement scheme allows for absolute distance measurement based on optical interferometry, without the need for displacement along a linear guidance. After a rough pre-measurement, spectral interferometry provides the absolute distance within an optical fringe, which can be further refined by utilizing the knowledge of individual wavelengths. In comparison to a counting helium-neon laser an agreement within an optical fringe has been demonstrated, which corresponds to an agreement within 10^−8^ for a distance of 50 m. In our setup the measurement uncertainty is dominated by environmental vibrations, prohibiting demonstration of the ultimate power of the homodyne interferometry. However, given the uncertainty on the determination of the phase an accuracy on the level of a few nm may be feasible in vacuum and a vibration-free environment. Under such conditions, e.g. in space, the measurement range could be extended to the level of km, with an accuracy far within an optical wavelength. The measurement range and best achievable accuracy will ultimately be limited by the coherence length of the light and thus the accuracy of the reference clock.

## Methods

### Measurement uncertainty

Several contributions to the measurement uncertainty can be distinguished. The length-dependent contributions are the uncertainty on the refractive index difference for both lasers, the repetition rate of the comb and the wavelength of the helium-neon laser. The standard uncertainty coming from refractive index is estimated to be <10^−9^. This is much smaller than the absolute knowledge of the refractive index, because for the comparison only the uncertainty coming from the refractive index *difference* between both wavelengths needs to be taken into account. The standard uncertainty of the HeNe laser is estimated to be <3 × 10^−9^, based on its frequency calibration and data acquisition system. The relative uncertainty on *f*_*rep*_ is typically 10^−11^ in 1 s and is estimated to be 10^−10^ in 10 ms. The uncertainty due to *f*_0_, which is only relevant for the homodyne interferometry methode, is negligible.

The distance-independent contributions to the measurement uncertainty are the uncertainty on the fitting parameters *C* and *D* and the uncertainty resulting from the read-out, including phase-interpolation, of the helium-neon laser. The standard uncertainty *u*_*C*_ on the fitting parameter *C* is found to be typically 10^−6^ < *u*_*C*_ < 10^−5^ for arbitrary distances and *u*_*C*_ < 10^−6^ for distances close to zero delay (*C* ≪ 1). For spectral interferometry this leads, via Eq. (12) to a contribution of 0.14 *μ*m to the uncertainty on the measured distance. The uncertainty of the helium-neon laser’s length-independent contributions, like phase-interpolation, is estimated to be < 0.01 *μ*m and can be neglected here.

For homodyne interferometry the uncertainty due to the fitting parameters is calculated via Eq. (13), using both the uncertainty on *C* and *D*. Here we find that the uncertainty doesn’t exceed 3 nm for all distances (<1 nm close to zero delay), for a wavelength *λ*_*p*_ in the middle of the spectrum (*p* − *D* = 3000). The sensitivity to the uncertainty on the fitting parameters is thus much smaller for homodyne interferometry, which is a direct consequence of *λ* ≪ Λ. This is a promising result for application of the presented method in quiet environments like space.

An important contribution to the comparison measurement, however, is caused by vibrations coupling to the carriage on the measurement bench. The data-acquisition times for the HeNe (0.1 s) and frequency comb (10 ms) are chosen to be optimal for the respective systems. This gives rise to sensitivity of the measurement to temporal fluctuations of the path length difference that occur on a timescale of 0.1 s. We have identified this contribution by a time-resolved measurement with the HeNe laser and found a typical contribution of environmental fluctuations to the measurement uncertainty of 0.22 *μ*m. This is the standard deviation of the temporal measurement with a sampling time of 0.1 s. Taking into account that these uncertainties occur at both the starting point and the end point of the measurement, the uncertainty due to environmental vibrations is estimated to be 0.31 *μ*m. To find the total measurement uncertainty for the spectral interferometry method at 50 m, this number is combined with the uncertainty of the fitting parameters (0.14 *μ*m) and the HeNe laser (0.15 *μ*m). The combined uncertainty as obtained from the quadratic sum of these contributions equals 0.37 *μ*m. For a coverage factor *k* = 2 (corresponding to a 95% coverage interval) the uncertainty equals 0.75 *μ*m. Clearly the uncertainty is dominated by fixed contributions, which is in agreement with the observations in Fig. 5. For homodyne interferometry the uncertainty due to the fitting parameters is negligible. This leads to an uncertainty on the comparison of 0.69 *μ*m (*k* = 2) at 50 m. The small difference between the uncertainty for spectral interferometry and homodyne interferometry again illustrates the dominant contribution of environmental vibrations in this comparison.

## Additional Information

**How to cite this article**: van den Berg, S. A. *et al.* Mode-resolved frequency comb interferometry for high-accuracy long distance measurement. *Sci. Rep.* **5**, 14661; doi: 10.1038/srep14661 (2015).
